# P-2210. Perinatal Acquisition of Resistant *E. coli* Alters the Gut Microbiome in a Murine Model

**DOI:** 10.1093/ofid/ofae631.2364

**Published:** 2025-01-29

**Authors:** Aspen N Kremer, Aspen N Kremer, Leena B Mithal, Alima Sajwani, Johanna Holm

**Affiliations:** Ann & Robert H. Lurie Children's Hospital, Chicago, Illinois; Ann & Robert H. Lurie Children's Hospital, Chicago, Illinois; Ann and Robert H. Lurie Children's Hospital of Chicago, Chicago, Illinois; Ann & Robert H. Lurie Children's Hospital, Chicago, Illinois; University of Maryland, Baltimore, Maryland

## Abstract

**Background:**

Maternal and neonatal colonization by pathogenic *E. coli*, particularly extended-spectrum beta-lactamase producing *E. coli* (ESBL-EC), heightens the risk of invasive infections in infants, complicating treatment and increasing morbidity. We developed a mouse model of perinatal transmission to explore this by first comparing the transmission rate among commensal *E. coli* strains, the pan-sensitive uropathogenic *E. coli* strain, UTI89, and four clinical ESBL-EC strains. We then determined the impact of early life acquisition of these strains on the mouse pup intestinal microbiome through sequencing analysis.

Figure 1

**Figure:**
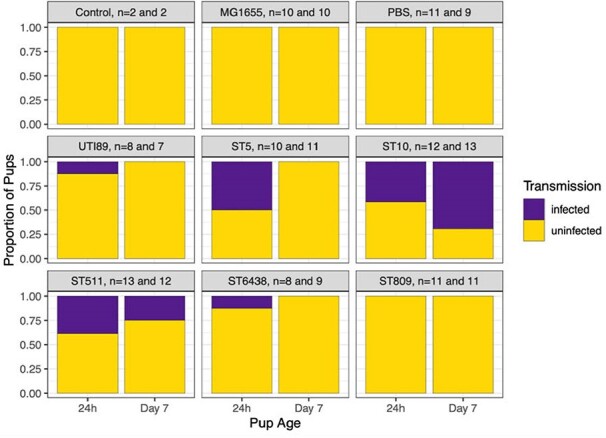

Proportion of pups colonized by each indicated strain, Sample sizes are for 24h and Day 7, respectively. The two control animals were not received no intervention.

**Methods:**

Pregnant dams were inoculated with control or test strains one week prior to expected delivery date. Fecal pellets were collected and plated on selective agar to determine burden of colonization. Pups were born without intervention and sacrificed 24h or 7 days later, large intestines were collected, and bacterial transmission was quantified using selective agar. DNA was extracted and the V3-V4 region of the 16s rRNA gene was amplified for next generation sequencing.

Figure 2

**Figure:**
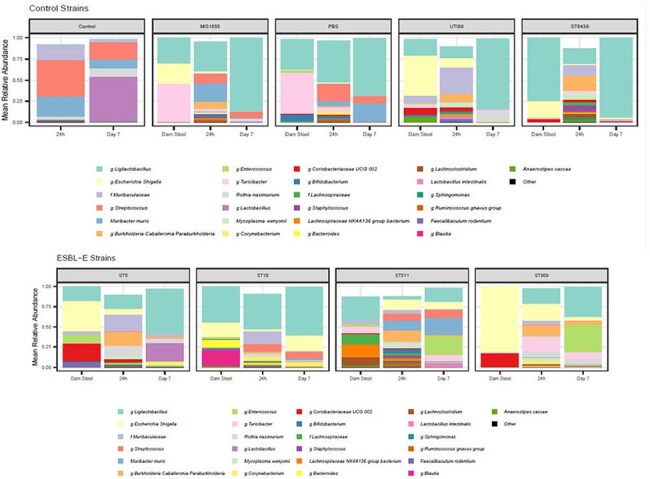

Microbiome analysis was conducted on dam fecal pellets and pup large intestines. Mean relative abundance of top 26 taxa is shown for dam stool before birth, and 24h and 7-day old pup large intestines. Control is no dam manipulation, MG1655 and ST6438 are non-pathogenic, pan-sensitive E. coli and UTI89 is a pan-sensitive uropathogenic E. coli strain.

**Results:**

While all strains adequately colonized the pregnant dam’s intestinal tract, only UTI89, ESBL-EC ST5, ST10, ST511, and the commensal ST6438 were transmitted to pups. ST511 and ST10 yielded colonization at both 24h and day 7 (Figure 1). Figure 2 shows the mean relative abundance of microbiota taxa in dam stool and pup intestines at 24h and 7 days old. As expected, pups born to dams colonized with *E. coli* had at least some *Escherichia* at 24h. However, at day 7, ESBL-EC ST10, ST511 and ST809 had higher *Escherichia* abundance than control strains, and all ESBL-EC strains had lower abundance of *Ligilactobacillus*, a common commensal, than the control strains. MG1655 and UTI89 pups had no *Escherichia* present by day 7. The ST511 pup data also suggests that ESBL-EC induced dysbiosis is not due to high maternal abundance and higher inoculation effect on pups, since *Escherichia* were not seen in a significant abundance in ST511 dams.

**Conclusion:**

These results suggest that *E. coli* strains differ in their ability to perinatally transmit, and that ESBL-EC strains more often cause a sustained dysbiosis of the murine gut. Our ongoing work aims to understand the impact of this early life dysbiosis on neonatal development.

**Disclosures:**

All Authors: No reported disclosures

